# Serplulimab plus chemotherapy as first-line treatment for extensive-stage small-cell lung cancer: A cost-effectiveness analysis

**DOI:** 10.3389/fimmu.2022.1044678

**Published:** 2023-01-04

**Authors:** Youwen Zhu, Kun Liu, Qun Qin, Hong Zhu

**Affiliations:** ^1^ Department of Oncology, Xiangya Hospital, Central South University, Changsha, Hunan, China; ^2^ National Institution of Drug Clinical Trial, Xiangya Hospital, Central South University, Changsha, Hunan, China; ^3^ National Clinical Research Center for Geriatric Disorders, Xiangya Hospital, Central South University, Changsha, Hunan, China

**Keywords:** extensive-stage small-cell lung cancer, serplulimab, etoposide and carboplatin, quality-adjusted life-years, incremental cost-effectiveness ratios

## Abstract

**Introduction:**

The ASTRUM-005 trial (NCT04063163) revealed that combination serplulimab plus chemotherapy (etoposide and carboplatin [EC]) treatment was associated with survival advantages relative to chemotherapy alone in patients diagnosed with extensive-stage small-cell lung cancer (ES-SCLC). As these immuno-chemotherapeutic regimens are extremely expensive, however, it is critical that the relative cost-effectiveness of combination serplulimab and chemotherapy treatment as a first-line treatment for ES-SCLC patients be examined in detail.

**Methods:**

The cost-effectiveness of combined serplulimab plus chemotherapeutic treatment was examined using a comprehensive Markov model with a 10-year boundary, enabling the calculation of overall cost, life years (LYs), quality-adjusted life-years (QALYs), and incremental cost-effectiveness ratio (ICER). Model instability was interrogated through one-way and probabilistic sensitivity analyses.

**Results:**

Serplulimab plus chemotherapy or chemotherapy alone respectively yielded 1.217 QALYs (2.243 LYs) and 0.885 QALYs (1.661 LYs) with corresponding total costs of $11,202 and $7,194, with an ICER of $12,077 per QALY ($6,883 per LY). This model was most strongly influenced by the utility of progression-free survival. Probabilistic sensitivity analysis showed that serplulimab plus chemotherapy had a 91.6% probability of being cost-effective at a willingness-to-pay (WTP) of $37,653 per QALY (3 × capita gross domestic product of China in 2021). In subgroup analyses, this combination treatment regimen was found to be most cost-effective in patients who were former smokers, had an ECOG performance status of 0, and were diagnosed with brain metastases.

**Conclusion:**

From a payer perspective in China, combination serplulimab plus chemotherapy treatment represents a cost-effective first-line intervention for ES-SCLC patients.

## 1 Introduction

Lung cancer is among the most common and deadliest cancers in China, with over 815,000 and 715,000 diagnoses and deaths, respectively, in 2021 alone (1). Small cell lung cancer (SCLC) is an aggressive subtype of neuroendocrine malignancy that accounts for 15% of total lung cnacer diagnoses. Strikingly, over 60% of SCLC patients are diagnosed after these tumors have already metastasized, and individuals with extensive-stage SCLC have an extremely poor 5-year survival rate of just 2% (2–5).

The advent of immunotherapeutic treatment regimens has contributed to significant improvements in ES-SCLC patient treatment options. The IMpower133 (NCT02763579) and CASPIAN (NCT03043872) studies initially demonstrated the value of combining etoposide and cisplatin/carboplatin (EP/EC)-based chemotherapeutic regimens with PD-L1 (programmed cell death 1 ligand 1) checkpoint inhibitor antibodies resulted in a more than 1-year increase in median patient overall survival (OS) and a 25% improvement in 2-year OS rate consistent with long-term benefit (6, 7). While these improvements are significant and represent a key step forward in the treatment of this deadly disease, further innovation is essential to yield satisfactory OS outcomes given that improved long-term survival is critical to provide patients with more opportunities for additional salvage treatment. The development of novel drugs and/or the establishment of optimized combination treatment regimens is vital to prolonging patient survival and to enhancing quality of life (QoL).

Serplulimab (Shanghai Fuhong Hanlin Biopharmaceutical Co. LTD) was a selective, structurally stable, a high-affinity recombinant humanized IgG4 monoclonal antibody against human programmed death protein 1(PD-1), which had a better anti-tumor effect because of the absence of antibody-dependent cell-mediated cytotoxicity (ADCC) and complement-dependent cytotoxicity (CDC) (8). Interim analyses of the randomized ASTRUM-005 clinical trial revealed a significant prolongation of the OS of patients treated with a combination of serplulimab plus chemotherapy relative to chemotherapy alone (15.4 *vs.*10.9 months; hazard ratio [HR], 0.63, 95% confidence interval [CI], 0.49 to 0.82; P < 0.001), with similar improvements in progression-free survival (PFS, 5.8 *vs.* 4.3 months; 0.47, 95% CI, 0.38 to 0.59; P < 0.001) (9). Relative to chemotherapy treatment, combination serplulimab plus chemotherapy regimens were also related to improvements in both OS (16.0 *vs.*11.1 months; HR, 0.62, 95% CI, 0.46 to 0.85; P = 0.002) and PFS (5.8 *vs.* 4.3 months; 0.45, 95% CI, 0.34 to 0.58; P < 0.001) among Asian populations (9). Subsequently, serplulimab plus chemotherapy was recommended (grade III, class IA evidence) as first-line treatment for patients with ES-SCLC by the Chinese Society of Clinical Oncology (CSCO Guidelines) in April 2021 (10). This application of serplulimab has been accepted by the National Medical Products Administration (NMPA) such that serplulimab is likely to become the first anti-PD-1 checkpoint inhibitor antibody in the world approved as a first-line option for individuals diagnosed with ES-SCLC.

While serplulimab has yielded unmatched improvements in ES-SCLC patient survival outcomes when combined with chemotherapy, the economic viability of immune checkpoint inhibitor (ICI)-based regimens and the populations in which such treatments are most economically beneficial remain to be established. The present study was thus designed to examine the cost-effectiveness of serplulimab plus chemotherapy as a first-line treatment for ES-SCLC patient treatment from the perspective of Chinese payers.

## 2 Methods

The consolidated health economic evaluation report standards statement (CHEERS) checklist was used to guide the design and execution of this study (**Supplementary Material eTable 1**).

### 2.1 Model structure and patient treatment

The cost-effectiveness of first-line treatment options for ES-SCLC patients were examined using a decision tree and a synthetic Markov model. Treatment options included in the decision tree included serplulimab plus chemotherapy and chemotherapy alone. **Supplementary Material eTable 2** provided comprehensive details regarding the administration and unit prices of the drugs included in this study. Three states were included in the structure of the Markov model: PFS, progressive disease (PD), and death (**Supplementary Material eFigure 1**). Patients began in the PFS state and underwent treatment with either of the included first-line regimens until PD or the discontinuation of treatment as a consequence of adverse events (AEs) or toxicity. After PD, subsequent treatment was administered to patients in both groups. Overall, Topotecan was subsequently administered as an antitumor regimen to 4.9% (19/389) and 4.1% (8/196) of patients in the serplulimab plus chemotherapy and chemotherapy groups, respectively (11, 12). All other patients were provided with the best supportive care (BSC), with terminal care being provided to individuals that experienced treatment-related mortality. For this study, included Chinese patients were presumed to be 61 years of age, to weigh 65 kg, to have a total body surface area of 1.72 m^2^, a serum creatinine level of 1 mg/dL, and an area under the curve (AUC) of 5 mg/mL/min (11, 13, 14).

In this study, the cycle length for the established Markov model was 6 weeks, with outcomes being developed with 10-year boundaries (More than 99% of patients die). A 3% annual discount rate was taken into account when modeling cost-effectiveness (15). Outputs of interest included total costs, life-years (LYs), quality-adjusted LYs (QALYs), and incremental cost-effectiveness ratio (ICER) values. The structure of this model and the data included therein were established based upon ASTRUM-005 trial results (11). The TreeAge Software (TreeAge Pro 2021; https://www.treeage.com) was used to design decision tree and Markov model analyses.

### 2.2 Survival estimates and model transitions

Data points were collected from PFS and OS curves using GetData (version 2.26; http://www.getdata-graph-digitizer.com/index.php), and these data were then used for parametric survival model fitting with Weibull, Exponential, Gompertz, Log-logistic, and Log-normal distributions. Based on Akaike’s and Bayseian information criteria, the Weibull distribution was selected. For further detail regarding the selection of these survival models, see **Supplementary Material eFigure 2** and **eTable 3**. When implementing Weibull distributions in R, a two-parameter model with estimated shape (γ) and scale (λ) was established based on such fitting and applied to Kaplan-Meier curves with R (version 4.0.2, http://www.r-project.org) and the method proposed by Hoyle (16) (**Table 1**).

**Table 1:** Model parameters: baseline values, ranges, and distributions for the sensitivity analysis.

Parameters	Baseline value	Range		Reference	Distribution
		Minimum	Maximum		
**Weibull survival model for OS of serplulimab plus chemotherapy**					
Overall population	Scale= 0.012874, Shape= 1.445565	–	–	(9)	–
Chinese	Scale= 0.009574, Shape= 1.535633	–	–	(9)	–
**Weibull survival model for PFS of serplulimab plus chemotherapy**					
Overall population	Scale= 0.14015, Shape= 0.92013			(9)	–
Chinese	Scale= 0.09846, Shape= 1.08898			(9)	–
**Weibull survival model for OS of chemotherapy**					
Overall population	Scale= 0.016452, Shape= 1.527988			(9)	–
Chinese	Scale= 0.03359, Shape= 2.07247			(9)	–
**Weibull survival model for PFS of chemotherapy**					
Overall population	Scale= 0.01472, Shape= 1.56003	–	–	(9)	–
Chinese	Scale= 0.03309, Shape= 2.15174	**-**	**-**	(9)	**-**
**Rate of post-discontinuation therapy**					
Chemotherapy group	0.049	0.039	0.059	(9)	Beta
Serplulimab plus chemotherapy group	0.041	0.033	0.049	(9)	Beta
**Risk for main AEs in chemotherapy group**					
Risk of anemia	0.054	0.043	0.065	(9)	Beta
Risk of thrombocytopenia	0.062	0.050	0.074	(9)	Beta
Risk of white blood cell decreased	0.085	0.068	0.102	(9)	Beta
Risk of decreased neutrophil count	0.141	0.113	0.169	(9)	Beta
**Risk for main AEs in serplulimab plus chemotherapy group**					
Risk of anemia	0.056	0.045	0.067	(9)	Beta
Risk of thrombocytopenia	0.082	0.066	0.098	(9)	Beta
Risk of white blood cell decreased	0.087	0.070	0.104	(9)	Beta
Risk of decreased neutrophil count	0.138	0.110	0.166	(9)	Beta
**Utility**					
Utility PFS	0.673	0.538	0.808	(17, 19)	Beta
Utility PD	0.473	0.378	0.568	(17, 19)	Beta
**Disutility**					
Anemia	0.074	0.059	0.089	(19)	Beta
Decreased neutrophil count	0.090	0.072	0.108	(19)	Beta
White blood cell decreased	0.090	0.072	0.108	(19)	Beta
Thrombocytopenia	0.200	0.160	0.240	(14)	Beta
**Drug cost, $/per cycle**					
Serplulimab	605	484	726	Local Charge	Gamma
Etoposide	12	10	14	Local Charge	Gamma
Carboplatin	73	58	88	Local Charge	Gamma
Topotecan	317	254	380	Local Charge	Gamma
**Cost of AEs, $**					
Chemotherapy	386	309	463	(14, 19)	Gamma
Serplulimab plus chemotherapy	309	247	371	(14, 19)	Gamma
**Administration per cycle**	36	29	43	(19)	Gamma
**Laboratory per cycle**	166	133	199	(19)	Gamma
**Tumor imaging per cycle**	507	406	608	(19)	Gamma
**Best supportive care per cycle**	221	177	265	(19)	Gamma
**Terminal care per patient**	2,221	1,777	2,665	(17)	Gamma
**Weight (Kg)**	65	52	78	(12)	Normal
**Body surface area (meters^2^)**	1.72	1.38	2.06	(12)	Normal
**Area under the curve (mg/mL/min)**	5	–	–	(12)	Uniform
**Serum creatinine (mg/dL)**	1	–	–	(13)	Uniform
**Discount rate**	0.03	–	–	(14)	Uniform

OS, overall survival; PFS, progression-free survival; PD, disease progressed; AEs, adverse events.

Time-dependency transition probabilities (tp) for the two patient treatment groups were extrapolated from ASTRUM-005 trial data, with tp values for each Markov cycle being calculated as follows: tp (tu)=1 − exp{λ(t − u)γ − λtγ} (λ > 0, γ > 0) (17). where u denotes the Markov cycle, and tu denotes the arrival at state t following u cycles.

### 2.3 Utility estimates

Utility values were used to approximate the QoL of patients, reflecting the impact of disease-related health on a scale from 0 (worst health) to 1 (optimal health). The mean health utility values for the PFS and PD states in this analysis were 0.673 and 0.473, respectively, based on published data (18, 19). Disutility values for AEs of grade 3 or higher were also taken into consideration in these analyses (15, 20).

### 2.4 Cost inputs

Factors considered when calculating direct medical costs included the costs of drugs, tumor imaging, laboratory testing, therapeutic administration, AE-related management, BSC, and terminal care. Drug costs were based on the mean sale prices in 2022 at the Xiangya Hospital of Central South University, while all other costs were based on published data sources (15, 18, 20). The model only took the costs of managing grade 3 or higher AEs and an AE frequency of > 5% into consideration (assuming AEs to only appear in one cycle of the PFS and PD states), with notably different probabilities between study arms (11). Follow-up costs included fees for magnetic resonance imaging or computed tomography scans performed every 6 weeks during treatment cycles from the date of randomization (11). All prices are expressed in US dollars, using the exchange rate $1 = ¥6.7584 (August 9, 2022).

### 2.5 Sensitivity analysis

Model result uncertainty was predicted with a range of sensitivity analyses. One-way sensitivity analyses were conducted within 20% of baseline values using different parameter values within defined ranges through the use of established approaches to assessing the effects of individual parameters on ICER values (21, 22). To conduct probabilistic sensitivity analyses, 10,000 Monte Carlo simulations were performed, enabling the simultaneous assessment of changes in several parameters (23). Cost-effectiveness acceptability curves for individual treatment strategies were evaluated the most cost-effective at the WTP threshold. All ASTRUM-005 trial patient subgroups were also taken into account. As OS curves were not provided for all of these subgroups, these curves were generated through the use of subgroup-specific HRs as per the methods reported by Zhu et al (15).

## 3 Results

### 3.1 Base case results

When taking QoL into account, this model projected that patients administered a combination serplulimab plus chemotherapy regimen would experience 1.217 QALYs (2.243 LYs), corresponding to 0.332 QALYs (0.582 LYs) more than for patients who only underwent chemotherapeutic treatment. Total costs associated with serplulimab plus chemotherapy and chemotherapy alone were $11,202 and $7,194, respectively, yielding ICERs of $12,077 per QALY ($6,883 per LY) relative to chemotherapy alone (**Table 2**).

**Table 2:** Baseline results.

Parameters	Serplulimab plus chemotherapy group	Chemotherapy group
LYs	2.243	1.661
QALYs	1.217	0.885
Total cost $	11,202	7,194
ICER $/LY	6,883 ^a^	NA
ICER $/QALY	12,077 ^a^	NA
WTP $/QALY	37,653	

^a^Compared to chemotherapy.

ICER, incremental cost-effectiveness ratio; LY, life-year; QALY, quality-adjusted life-year; WTP, willingness-to-pay.

### 3.2 Sensitivity analysis

**Figure 1:**
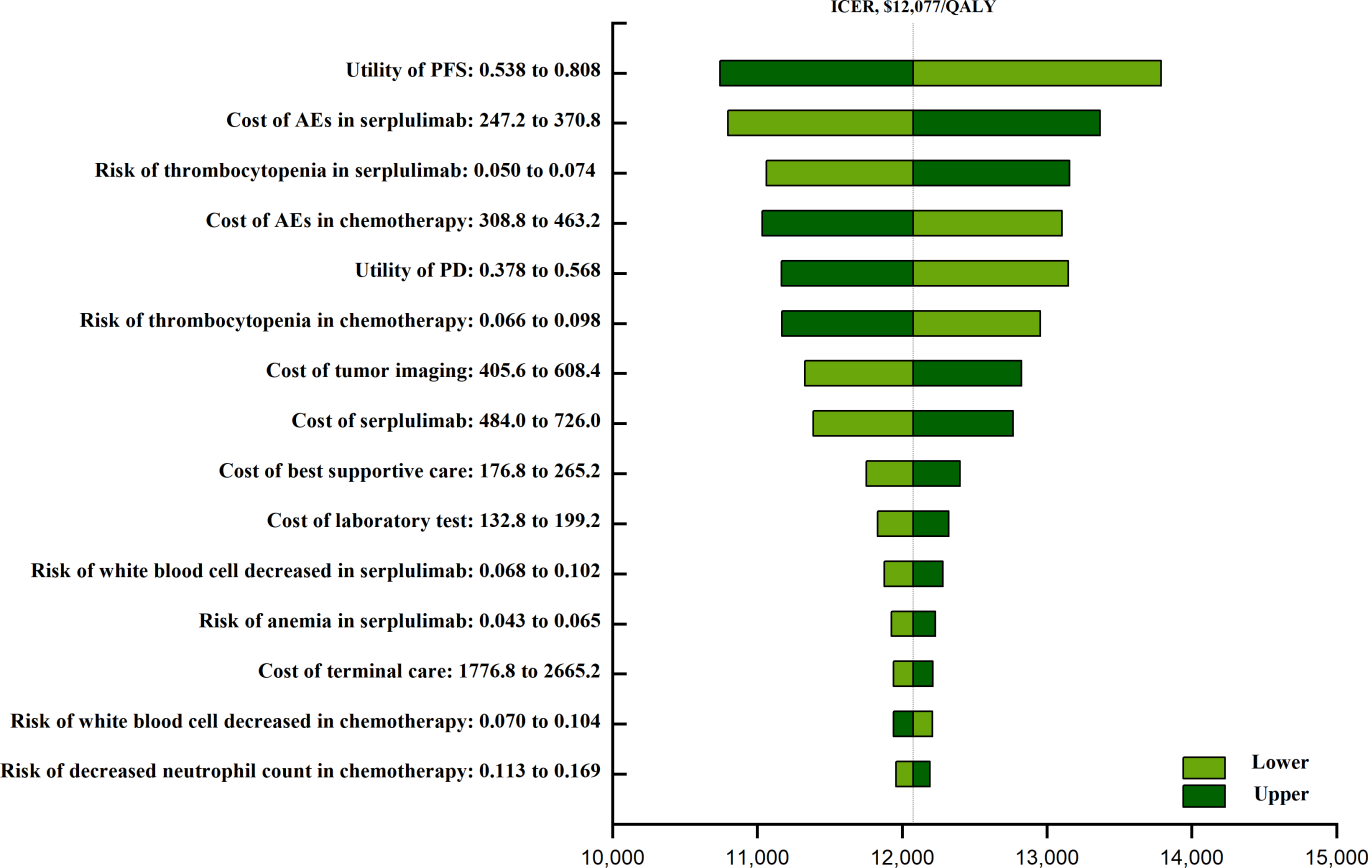
The one-way sensitivity analyses of serplulimab plus chemotherapy group and chemotherapy group. PFS, progression-free survival; AEs, adverses events; PD, progressive disease.

The one-way sensitivity analyses (**Figure 1**) revealed that that the factor that had the greatest impact on ICER values was the utility of PFS (ranging from 0.538 to 0.808, with the ICER increasing from $ 10,743 to $ 13,788 per QALY), followed by the costs associated with AE treatment in patients undergoing serplulimab plus chemotherapy treatment, the risk of thrombocytopenia in this combination treatment group, and the cost of AE treatment in the chemotherapy group. The actual costs of chemotherapy and AE disutility values had little impact on these results.

**Figure 2:**
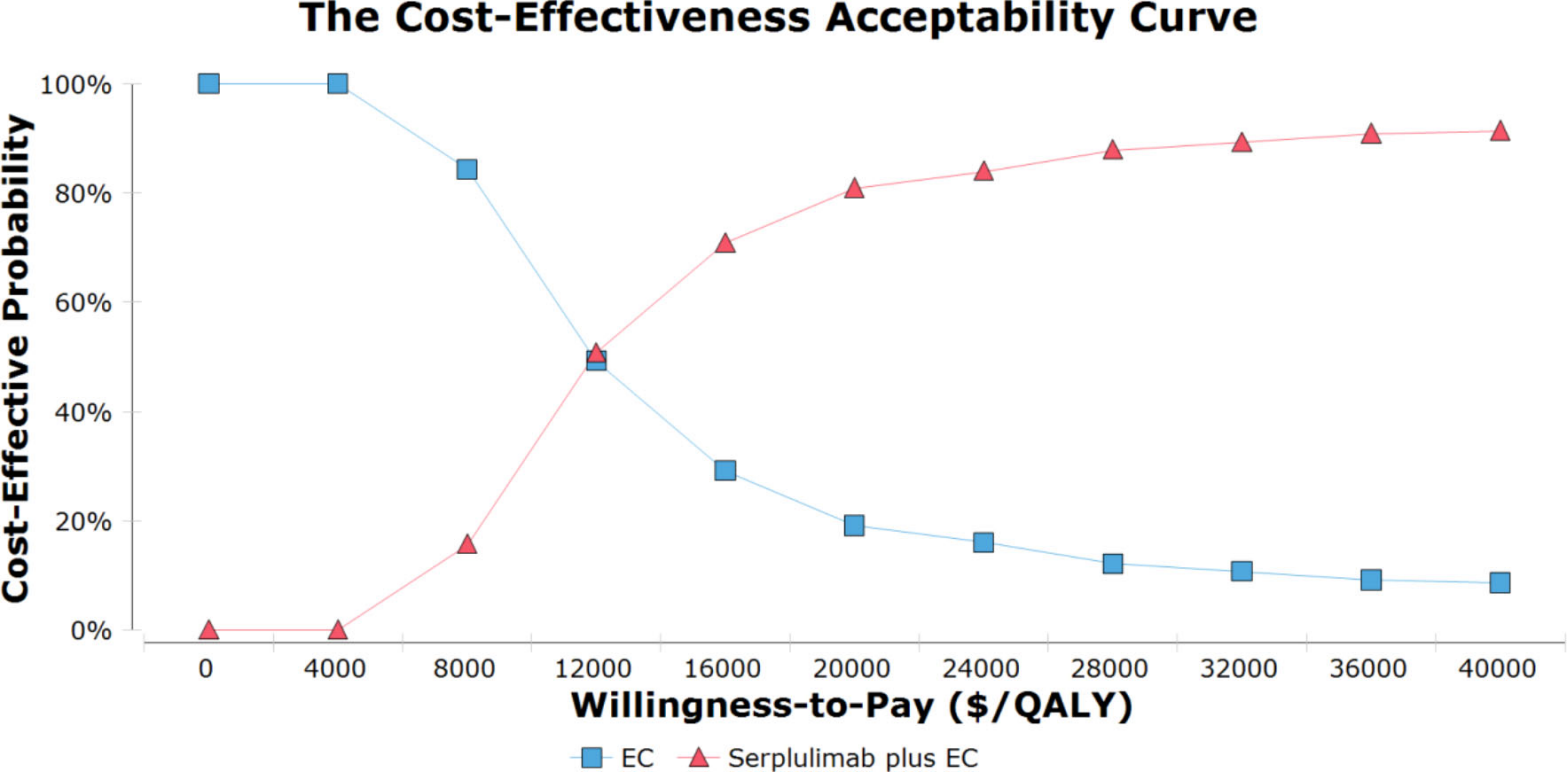
The cost-effectiveness acceptability curves for serplulimab plus chemotherapy group compared to chemotherapy group. EC, etoposide and carboplatin; QALY, quality-adjusted life-year.

Probabilistic sensitivity analysis results are given as acceptable curves (**Figure 2**) and scatterplots (**Supplementary Material eFigure 3**). The odds of serplulimab plus chemotherapy being cost-effective relative to chemotherapy along at a WTP threshold of $37,653 per QALY was 91.6%. Acceptability curves indicated that the odds of this combined treatment regimen being cost-effective rose with increasing WTP threshold values.

Subgroup analyses indicated that serplulimab plus chemotherapy treatment was associated with the prolongation of patient OS relative to chemotherapy alone, with ICERs for this comparison ranging from $9,128 to $30,386 per QALY. In probabilistic sensitivity analyses, serplulimab plus chemotherapy treatment exhibited greater cost-effectiveness in patients with ECOG performance status of 0 (94.1%), former smokers (92.9%), and patients diagnosed with brain metastases (92.9%) (**Supplementary Material eTable 4**).

## 4 Discussion

Total direct medical expenditures for lung cancer in China in 2017 were $10.3 billion, corresponding to 0.0497% of total GDP (24). This economic burden is forecast to rise to $30.1 billion, $40.4 billion, and $53.4 billion in 2020, 2025, and 2030, respectively (24). Given these rising overall expenditures and the limitations to medical resource access facing lung cancer patients in China, there is a growing focus on the need for value-based oncological treatment. Economic evaluations offer a simple and theoretically informed means of gauging the costs and outcomes associated with particular treatment regimens while handling uncertainty and dealing with social and individual choices (25). Significant positive outcomes were reported in the phase III randomized ASTRUM-005 trial when comparing the relative safety and efficacy of first-line serplulimab plus chemotherapy to chemotherapy alone among ES-SCLC patients. This is a major advance in the treatment of this patient population. Accordingly, a Markov model was herein used to examine the cost-effectiveness of such treatments from the perspective of Chinese payers, comparing the relative costs and efficacy of chemotherapy with and without serplulimab at a WTP of $37.653 per QALY.

The development of combination ICI-based treatment approaches has marked a major shift in the management of ES-SCLC patients (6, 7, 26). The development of combination ICI-based treatment approaches has marked a major shift in the management of ES-SCLC patients (6, 7, 26). When analyzing data from three trials, Zhou et al., Ding et al., and Zhu et al. found combination chemotherapy and atezolizumab, durvalumab, or pembrolizumab treatment to yield respective ICERs of $528,810, $464,711.90, and $647,509 per QALY in the US as compared to chemotherapy alone (12, 20, 27). Li et al. and Liu et al. also found that ICERs in China associated with combining chemotherapy and atezolizumab or durvalumab were $489,013 and $192,591 per QALY, respectively (28, 29). These results consistently support the fact that favorable ICI-related survival benefits observed in clinical trials correspond to poor ICI cost-effectiveness in both developed and developing countries, primarily owing to the high costs associated with these ICI regimens. It is vital that doctors and managers remain aware when seeking to select therapeutic options with a high performance-price ratio when guiding patient care in the context of innovative drug treatment, with the overall goal of supporting healthcare sustainability.

The results of this study suggest that serplulimab plus chemotherapy is more cost-effective than EC chemotherapy alone when used as a first-line treatment option for ES-SCLC patients, with an ICER of $12,077 per QALY, well below the WTP in China of $37,653 per QALY. Additional costs associated with this combination therapeutic regimen were primarily attributable to the costs of care and to AE incidence, emphasizing the importance of reducing AE incidence to the greatest extent possible. Sensitivity analyses did not reveal any changes in these conclusions with variations in these parameters, supporting the robustness of this mode. One-way sensitivity analyses additionally revealed that economic outcomes associated with ICI-based treatment were likely to be more favorable for patients with lower utility, while declining when higher utility levels are evident. This is in contrast with prior studies in which ICI prices were found to have the highest impact on cost-effectiveness outcomes for these regimens in ES-SCLC patient populations (12, 27, 29–34). This may be the result of the charitable aid available for serplulimab in China (buy 6 for free, then buy 6 for 2 years), supporting the improved cost efficiency of serplulimab plus chemotherapy in ES-SCLC patients.

Patients exhibiting a baseline PD-L1 tumor proportion score (TPS) < 1% presented with longer median OS relative to patients with a PD-L1 TPS ≥ 1% in both treatment groups in this study. Further consideration of whether serplulimab plus chemotherapy was more cost-effective than EC chemotherapy alone in these patients thus warranted further consideration such that baseline PD-L1 TPS values were taken into consideration when performing subgroup analyses. This approach ultimately supported the cost-effectiveness of combined serplulimab plus chemotherapy treatment in all tested subgroups. In their prior retrospective analysis, Ishii et al. did not observe any significant correlation between the extensive disease (ED) stage and OS (35). However, a specific focus on patients with ES-SCLC revealed the prolongation of median OS among patients positive for PD-L1 expression relative to patients that were PD-L1-negative (9.2 months *vs.* 5.4 months, P = 0.037), whereas no significant difference in median PFS was evident between these groups (5.2 months *vs.* 4.6 months, P = 0.747) (35). PD-L1 status may thus be a suboptimal biomarker when planning anti-PD-1/PD-L1 therapy. However, it is important to note that further detailed analyses of the factors influencing ICERs in these subgroups are warranted, particularly as only OS HRs were taken into consideration and stratification was only performed for two PD-L1 TPS subsets. Caution is thus necessary when interpreting these results.

Widespread concern regarding the affordability of and access to cancer treatments is currently shared by both patients and clinicians. Therapeutic regimens that entail high out-of-pocket costs despite minimal improvement in oncology patient outcomes undermine the goal of providing patients with lifesaving high-quality care. With the continued expansion of cancer treatment options and the development of combined immunochemotherapeutic regimens, it is critical that clinicians remain aware of both the clinical and economic advantages associated with particular treatment strategies in order to address the financial burdens that face patients as a consequence of oncological care (36). These results offer evidence that Chinese payers can use to mitigate potential financial toxicity *via* suggesting the most cost-effective immunotherapeutic treatment options for ES-SCLC patients.

There are certain limitations to this study that require caution when interpreting these findings. As these analyses were based on the ASTRUM-005 trial given that it is the only randomized phase III trial to date comparing the first-line treatment of ES-SCLC patients with chemotherapy with or without serplulimab, any biases inherent in that trial will impact the results of this study. The ASTRUM-005 trial OS data remain immature at present, further potentially constraining the present analyses. Moreover, the limited clinical data that were available from the ASTRUM-005 trial were extrapolated in Markov model analyses to gauge long-term patient outcomes such that the results will inevitably be subject to some degree of uncertainty. When mature OS data from this and other clinical trials are available, this model can be further validated. As Kaplan-Meier curves for patient subgroups were not provided by the ASTRUM-005 trial, it was not possible to fully optimize this model for all subgroups of interest, and these subgroups may not exhibit the level of balance present in the original patient population as a result of randomization. Caution is thus warranted when interpreting subgroup analysis results. Moreover, costs associated with grade 1/2 AEs or immune-related AEs were not taken into consideration in this study, potentially contributing to the overestimation of the benefits of serplulimab plus chemotherapy treatment. Even so, these AEs are generally considered controllable with standard monitoring, and are not markedly associated with QoL (37). As is the case for most modeling analyses, no prospective data collection was performed herein, and these data may thus not accurately reflect the true clinical situation for the patient population of interest. Finally, our study is based on the innovative PD-1 drug (serplulimab) independently developed in China, which is difficult to promote to other countries’s healthcare systems in a short time due to whether the drug is allowed to be marketed in foreign countries, but it is of great significance in China’s healthcare system and clinical practice.

## 5 Conclusion

According to our study, serplulimab plus chemotherapy is the first immune-chemotherapy regimen that is highly likely to represent a better trade-off than chemotherapy alone for patients with ES-SCLC in the first-line setting at a WTP threshold of $37,653 per QALY. This finding may help China’s healthcare decision-making and medical reimbursement policy formulation.

## Data availability statement

The original contributions presented in the study are included in the article/**Supplementary Material**. Further inquiries can be directed to the corresponding authors.

## Author contributions

YWZ, KL, and HZ designed the experiment. YWZ and KL performed the experiments. YWZ and KL analyzed the data. QQ, and HZ contributed analysis tools and funding. YWZ, KL, QQ, and HZ wrote the manuscript. HZ and QQ are co-corresponding authors. All authors have read and approved the manuscript.
