# Supplementary material for: Serplulimab plus chemotherapy as first-line treatment for extensive-stage small-cell lung cancer: A cost-effectiveness analysis
Source: Front Immunol. 2023 Jan 4;13:1044678. doi: 10.3389/fimmu.2022.1044678 (PMC9845593; doi:10.3389/fimmu.2022.1044678)
Supplement: Supplementary file 1 [file DataSheet_1.pdf]

eFigure 1. Model Structure.

eFigure 2. Kaplan-Meier Curve Fitting and Extrapolation.

eFigure 3. Probability Sensitivity Analysis Scatter Plot.

eTable 1. CHEERS Checklist.

eTable 2. Drug Dose and Cost.

eTable 3. Summary of Ctatistical Goodness-of-fit of K-M Curve.

eTable 4. Results of Subgroup Analyses.

eFigure 1. Model Structure.

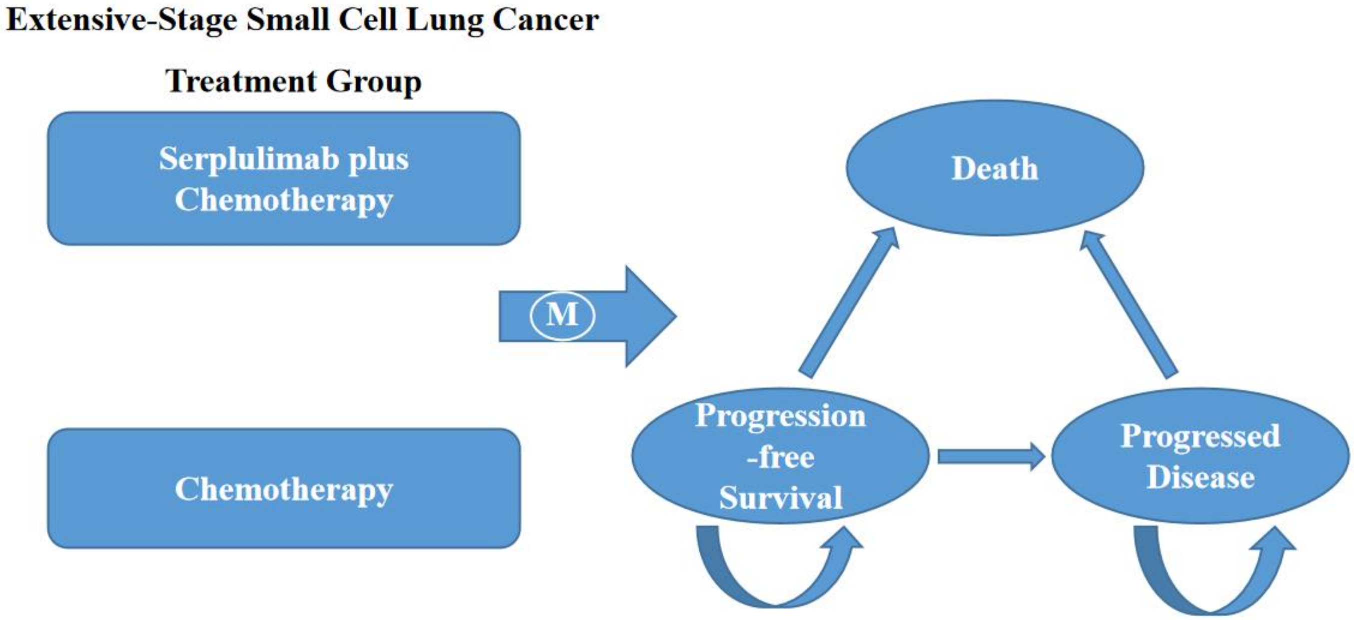

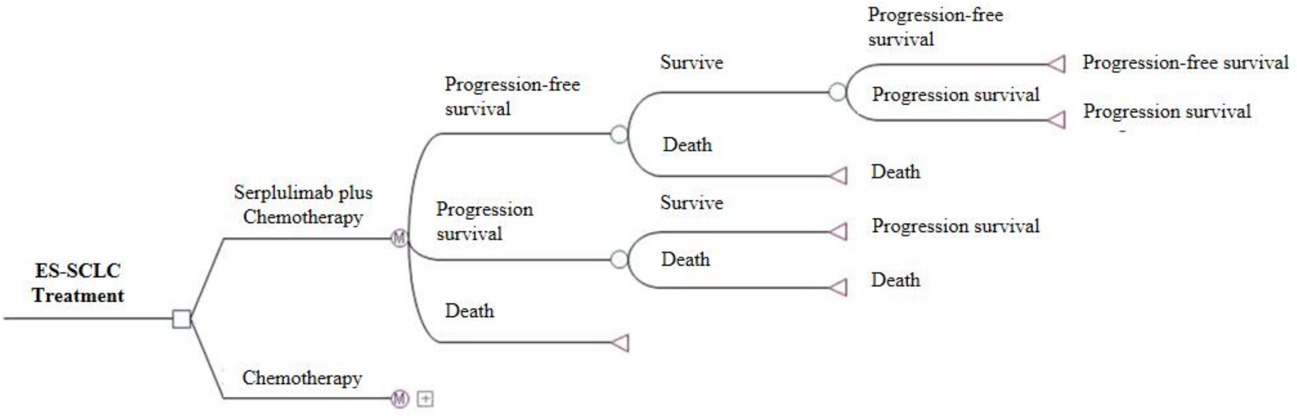

Abbreviation: M, Markov; ES-SCLC, extensive-stage small cell lung cancer.

eFigure 2. Kaplan-Meier Curve Fitting and Extrapolation.

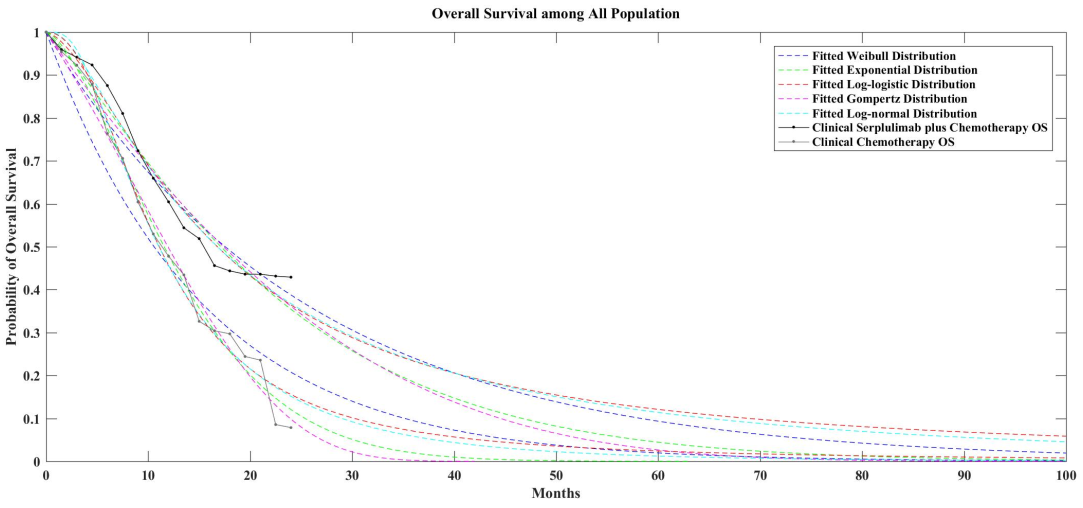

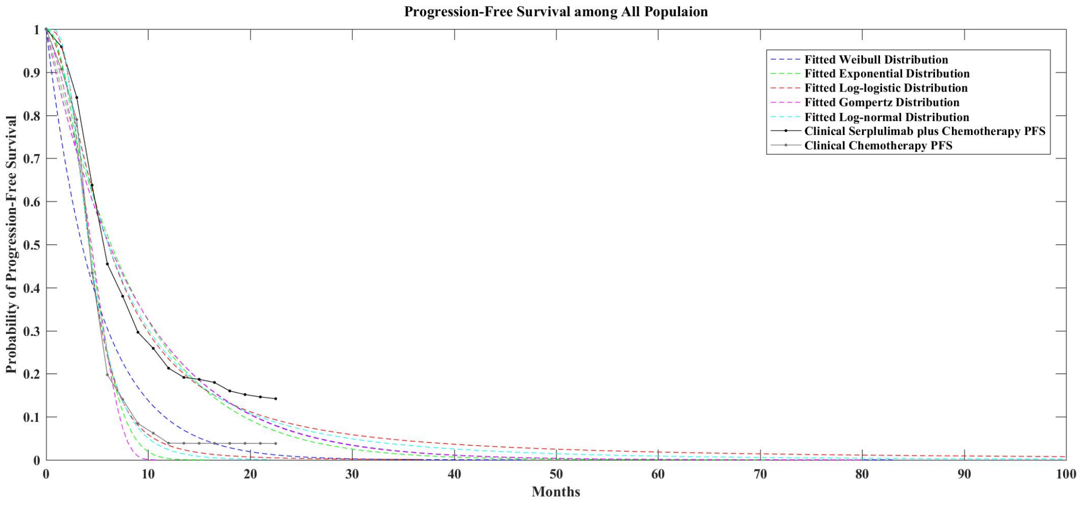

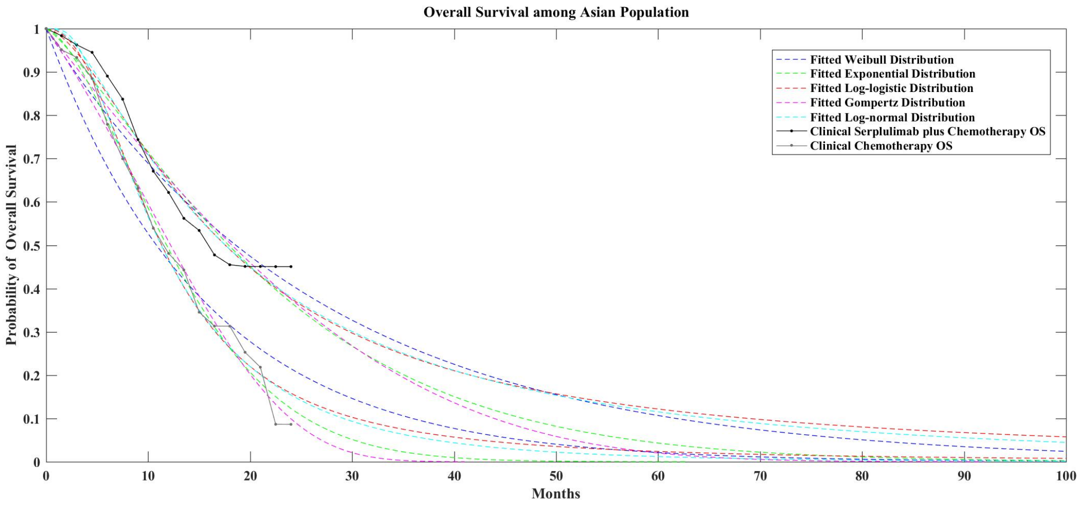

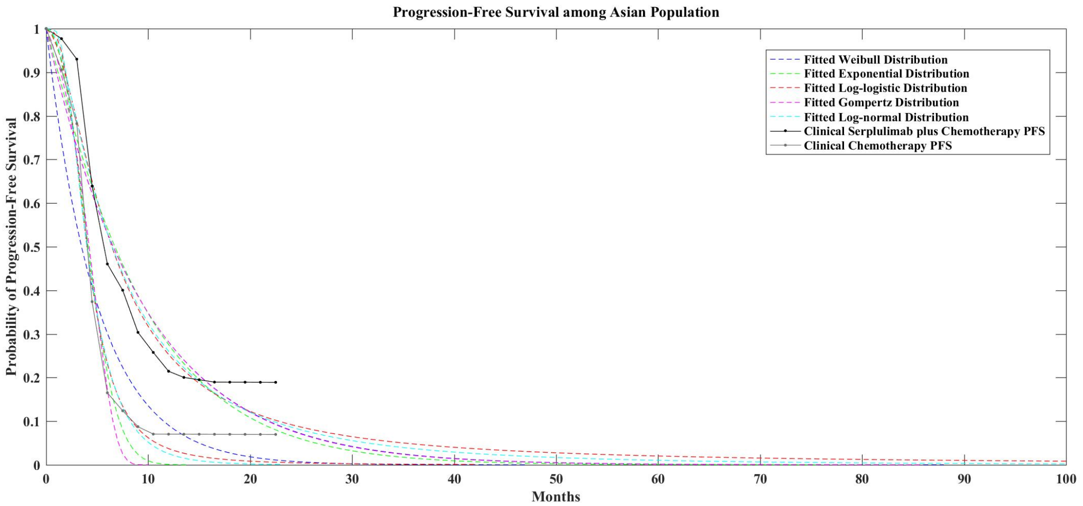

Abbreviation: T-DXd, trastuzumab deruxtecan; OS, overall survival; PFS, progression-free survival.

eFigure 3. Probability Sensitivity Analysis Scatter Plot.

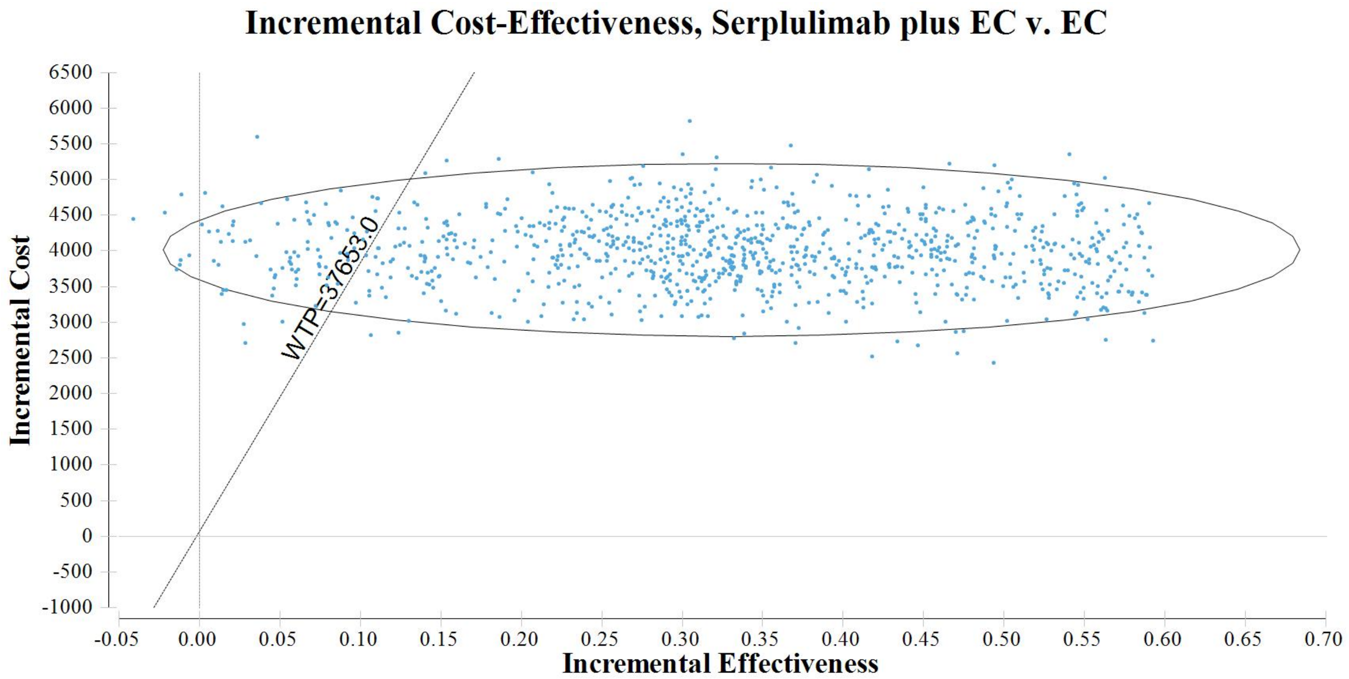

Abbreviation: EC, etoposide and carboplatin; WTP, willingness-to-pay.

Each point in the diagram represents a simulation result of 10,000 Monte Carlo simulation. Ellipse represent the 95% CI and dotted line represent WTP of $37,653/QALY. Points to the right of the dotted line are considered cost-effective.

eTable 1. CHEERS Checklist.

Section/item	Item No	Recommendation	Reported on page No
**Title and abstract**			
Title	1	Identify the study as an economic evaluation or use more specific terms such as "cost-effectiveness analysis", and describe the interventions compared.	I
Abstract	2	Provide a structured summary of objectives, perspective, setting, methods (including study design and inputs), results (including base case and uncertainty analyses), and conclusions.	II
**Introduction**			
Background and objectives	3	Provide an explicit statement of the broader context for the study.	1, 2
		Present the study question and its relevance for health policy or practice decisions.	
**Methods**			
Target population and subgroups	4	Describe characteristics of the base case population and subgroups analysed, including why they were chosen.	2, 3, 5
Setting and location	5	State relevant aspects of the system(s) in which the decision(s) need(s) to be made.	2, 3
Study perspective	6	Describe the perspective of the study and relate this to the costs being evaluated.	3-5
Comparators	7	Describe the interventions or strategies being compared and state why they were chosen.	2, 3
Time horizon	8	State the time horizon(s) over which costs and consequences are being evaluated and say why appropriate.	3
Discount rate	9	Report the choice of discount rate(s) used for costs and outcomes and say why appropriate.	3
Choice of health outcomes	10	Describe what outcomes were used as the measure(s) of benefit in the evaluation and their relevance for the type of analysis performed.	3
Measurement of effectiveness	11a	*Single study-based estimates:* Describe fully the design features of the single effectiveness study and why the single study was a sufficient source of clinical effectiveness data.	3, 4

	11b	*Synthesis-based estimates:* Describe fully the methods used for identification of included studies and synthesis of clinical effectiveness data.	
Measurement and valuation of preference based outcomes	12	If applicable, describe the population and methods used to elicit preferences for outcomes.	Not applicable
Estimating resources and costs	13a	*Single study-based economic evaluation:* Describe approaches used to estimate resource use associated with the alternative interventions. Describe primary or secondary research methods for valuing each resource item in terms of its unit cost. Describe any adjustments made to approximate to opportunity costs.	5
	13b	*Model-based economic evaluation:* Describe approaches and data sources used to estimate resource use associated with model health states. Describe primary or secondary research methods for valuing each resource item in terms of its unit cost. Describe any adjustments made to approximate to opportunity costs.	
Currency, price date, and conversion	14	Report the dates of the estimated resource quantities and unit costs. Describe methods for adjusting estimated unit costs to the year of reported costs if necessary. Describe methods for converting costs into a common currency base and the exchange rate.	5
Choice of model	15	Describe and give reasons for the specific type of decision-analytical model used. Providing a figure to show model structure is strongly recommended.	2, 3<br>eFigure 1
Assumptions	16	Describe all structural or other assumptions underpinning the decision-analytical model.	3-5
Analytical methods	17	Describe all analytical methods supporting the evaluation. This could include methods for dealing with skewed, missing, or censored data; extrapolation methods; methods for pooling data; approaches to validate or make adjustments (such as half cycle corrections) to a model; and methods for handling population heterogeneity and uncertainty.	3, 4<br>eFigure 2<br>eTable 3
**Results**			

Study parameters	18	Report the values, ranges, references, and, if used, probability distributions for all parameters. Report reasons or sources for distributions used to represent uncertainty where appropriate. Providing a table to show the input values is strongly recommended.	2-5<br>Table 1
Incremental costs and outcomes	19	For each intervention, report mean values for the main categories of estimated costs and outcomes of interest, as well as mean differences between the comparator groups. If applicable, report incremental cost-effectiveness ratios.	5, 6<br>Table 2
Characterising uncertainty	20a	*Single study-based economic evaluation:* Describe the effects of sampling uncertainty for the estimated incremental cost and incremental effectiveness parameters, together with the impact of methodological assumptions (such as discount rate, study perspective).	5, 6
	20b	*Model-based economic evaluation:* Describe the effects on the results of uncertainty for all input parameters, and uncertainty related to the structure of the model and assumptions.	
Characterising heterogeneity	21	If applicable, report differences in costs, outcomes, or cost-effectiveness that can be explained by variations between subgroups of patients with different baseline characteristics or other observed variability in effects that are not reducible by more information.	Not applicable
**Discussion**			
Study findings, limitations, generalisability, and current knowledge	22	Summarise key study findings and describe how they support the conclusions reached. Discuss limitations and the generalisability of the findings and how the findings fit with current knowledge.	6-10
**Other**			
Source of funding	23	Describe how the study was funded and the role of the funder in the identification, design, conduct, and reporting of the analysis. Describe other non-monetary sources of support.	11
Conflicts of interest	24	Describe any potential for conflict of interest of study contributors in accordance with journal policy. In the absence of a journal policy, we recommend authors comply with International Committee of Medical Journal Editors recommendations.	11

A good template page for CHEERS Checklist is as follows: http://www.ispor.org/TaskForces/EconomicPubGuidelines.asp.

Reference:

Husereau D, Drummond M, Petrou S, et al. Consolidated health economic evaluation reporting standards (CHEERS) —Explanation and elaboration: A report of the ISPOR health economic evaluations publication guidelines good reporting practices task force. Value Health 2013;16:231-50.

eTable 2. Drug Dose and Cost.

Drug	Dose	Time	Unit costs ($/mg)
Serplulimab plus chemotherapy	Serplulimab, 4.5 mg/kg	Four 3-week cycles; followed by maintenance serplulimab every 3 weeks (a maximum of 2 years of treatment)	1.0335
	Etoposide, 100 mg/m^2 on days 1-3		0.0115
	Carboplatin, area under curve 5		0.0761
Chemotherapy	Etoposide, 100 mg/m^2 on days 1-3	Four 3-week cycles; followed by maintenance placebo every 3 weeks (a maximum of 2 years of treatment)	0.0115
	Carboplatin, area under curve 5		0.0761
Topotecan	Topotecan, 1.25 mg/m^2, on day 1-5	Administered intravenously every 3 weeks	14.7224

eTable 3. Summary of Statistical Goodness-of-fit of K-M Curve.

	Exponential	Weibull	Gompertz	Log-logistic	Log-normal
**Overall population**					
**Serplulimab plus Chemotherapy OS curve**					
AIC	20.1487	18.1109	20.1622	20.1154	20.0282
BIC	21.8151	18.9441	21.8286	21.7818	21.6947
**Chemotherapy OS curve**					
AIC	32.9101	28.7233	33.5271	31.9868	32.0282
BIC	34.5766	29.5565	35.1936	33.6532	33.6946
**Serplulimab plus Chemotherapy PFS curve**					
AIC	45.7676	42.4282	44.5098	44.5780	44.6074
BIC	47.3128	43.2007	46.0550	46.1232	46.1525
**Chemotherapy PFS curve**					
AIC	233.4982	73.3239	Infinite	95.5516	112.9789
BIC	235.0434	74.0965	Infinite	97.0968	114.5241
**Asian population**					
**Serplulimab plus Chemotherapy OS curve**					
AIC	19.2338	17.2075	19.2547	19.2216	19.1351
BIC	20.9002	18.0408	20.9211	20.8880	20.8015
**Chemotherapy OS curve**					
AIC	32.3155	28.1513	32.9612	31.412	31.4533
BIC	33.9819	28.9845	34.6276	33.0784	33.1197
**Serplulimab plus Chemotherapy PFS curve**					
AIC	43.1002	39.9915	42.586	42.5860	42.4510
BIC	44.6454	40.7641	44.1312	44.1312	43.9962

**Chemotherapy PFS curve**					
AIC	285.8387	73.9620	Infinite	92.8577	109.8266
BIC	287.3839	74.7346	Infinite	94.4029	111.3718

Abbreviation: OS, overall survival; PFS, progression-free survival; AIC, Akaike's information criterion; BIC, Bayesian information criterion.

According to the AIC and BIC value of the distributions and the visual fitting of the curves (eFigure 2) showed that the Weibull distribution is probably the most reasonable parametric survival model. Weibull distribution is flexible and widely used for matching patients with three states over time, because it can monotonously increase or decrease risk functions, and it is suitable for estimating events occurring during early follow-up work.

eTable 4. Results of Subgroup Analyses.

Subgroup	No. of patients		OS HR (95% CI)	ICER, $/QALY	Cost-effectiveness probability of serplulimab plus EC at WTP, %
	Serplulimab plus EC	EC			$3,7653/QALY
**Age, years**					
＜ 65	89	60	0.62 (0.45 to 0.86)	12,031	92.2
≥ 65	57	40	0.60 (0.40 to 0.89)	11,615	92.5
**Sex**					
Male	121	84	0.64 (0.48 to 0.84)	12,483	90.1
Female	25	16	0.57 (0.30 to 1.06)	11,046	92.8
**ECOG performance status**					
0	21	16	0.44 (0.23 to 0.84)	9,128	94.1

1	125	84	0.65 (0.49 to 0.86)	12,724	91.6
**Smoking status**					
Current	37	24	0.61 (0.36 to 1.02)	11,819	91.9
Former	75	58	0.59 (0.42 to 0.83)	11,418	92.9
Never	34	18	0.75 (0.42 to 1.33)	15,891	82.8
**Baseline brain metastasis**					
No	123	82	0.62 (0.47 to 0.82)	12,031	92.2
Yes	23	18	0.61 (0.33 to 1.33)	11,819	92.9
**PD-L1 TPS**					
＜1%	121	83	0.58 (0.44 to 0.76)	11,229	92.0
⩾1%	22	11	0.92 (0.44 to 1.89)	30,386	63.6
Unknown	3	6	0.42 (0.10 to 1.72)	NA[a]	NA[a]

[a] The sample size was too small and was not further calculated.

Abbreviation: CI, confidence interval; ICER, incremental cost-effectiveness ratio; LY, life-year; OS HR, overall survival hazard ratio; PD-L1, programmed cell death-Ligand 1; TPS, Tumor Proportion Score; QALY, quality-adjusted life-year; WTP, Willingness-to-pay.
